# A machine-learning model to predict postoperative delirium following knee arthroplasty using electronic health records

**DOI:** 10.1186/s12888-022-04067-y

**Published:** 2022-06-27

**Authors:** Jong Wook Jung, Sunghyun Hwang, Sunho Ko, Changwung Jo, Hye Youn Park, Hyuk-Soo Han, Myung Chul Lee, Jee Eun Park, Du Hyun Ro

**Affiliations:** 1 Department of Orthopedic Surgery, Seoul National University College of Medicine, Seoul, South Korea; 2 Department of Orthopedic Surgery, Seoul National University Hospital, 101 Daehak-ro, Jongno-gu, Seoul, 110-744 South Korea; 3 Department of Psychiatry, Seoul National University Bundang Hospital, Seoul, South Korea; 4 Department of Psychiatry, Seoul National University Hospital, Seoul, South Korea; 5 CONNECTEVE Co., LTD., Seoul, South Korea

**Keywords:** Delirium, Total knee arthroplasty, Machine learning, Prediction, Neurologic disorder, Preoperative model

## Abstract

**Background:**

Postoperative delirium is a challenging complication due to its adverse outcome such as long hospital stay. The aims of this study were: 1) to identify preoperative risk factors of postoperative delirium following knee arthroplasty, and 2) to develop a machine-learning prediction model.

**Method:**

A total of 3,980 patients from two hospitals were included in this study. The model was developed and trained with 1,931 patients from one hospital and externally validated with 2,049 patients from another hospital. Twenty preoperative variables were collected using electronic hospital records. Feature selection was conducted using the sequential feature selection (SFS). Extreme Gradient Boosting algorithm (XGBoost) model as a machine-learning classifier was applied to predict delirium. A tenfold-stratified area under the curve (AUC) served as the metric for variable selection and internal validation.

**Results:**

The incidence rate of delirium was 4.9% (*n* = 196). The following seven key predictors of postoperative delirium were selected: age, serum albumin, number of hypnotics and sedatives drugs taken preoperatively, total number of drugs (any kinds of oral medication) taken preoperatively, neurologic disorders, depression, and fall-down risk (all *p* < 0.05). The predictive performance of our model was good for the developmental cohort (AUC: 0.80, 95% CI: 0.77–0.84). It was also good for the external validation cohort (AUC: 0.82, 95% CI: 0.80–0.83). Our model can be accessed at https://safetka.connecteve.com.

**Conclusions:**

A web-based predictive model for delirium after knee arthroplasty was developed using a machine-learning algorithm featuring seven preoperative variables. This model can be used only with information that can be obtained from pre-operative electronic hospital records. Thus, this model could be used to predict delirium before surgery and may assist physician’s effort on delirium prevention.

**Supplementary Information:**

The online version contains supplementary material available at 10.1186/s12888-022-04067-y.

## Background

The prevalence of elective knee arthroplasty for arthritis continues to increase, reaching 1.5% in the general population and 10.4% in those aged 80 years [1, 2]. A pattern of increasing frequency has been reported by many worldwide joint registries [2–4]. Knee arthroplasty is being extended to patients who are older than 60 years, or those who have substantially comorbidities, or those who have preoperative symptoms [2]. However, systemic complications such as deep vein thrombosis, delirium, and renal complications can occur in 6–9% of patients who undergo knee arthroplasty, especially in older patients [5, 6]. As the number of knee arthroplasty in older patients and those who have comorbidities is increasing annually, it is preferrable to prevent postoperative complications [6]. Of these complications, postoperative delirium can result in significant delay in rehabilitation, prolonged hospitalization, and increased mortality [4]. The incidence of delirium after elective joint arthroplasty, including knee arthroplasty, is 5–17% [5, 7, 8]. Despite its potential adverse effects, there is no consensus with regard to prevailing risk factors of delirium for developing prevention strategies [6].

Electronic health records (EHRs) accumulate huge amounts of data, facilitating machine-learning and the use of artificial intelligence [9–12]. Machine-learning models for prediction of delirium have also been developed. These models have performed better than logistic regression models for hospitalized patients [10, 12–14]. However, these models don’t target knee arthroplasty patients. In addition, these models use unmodifiable data such as age [10, 14], female [14], number of diagnosis [10], and data that can be collected only retrospectively such as length of hospital stay [10]. Moreover, all these models were based on data from single hospitals without external validation. It remains unclear whether these machine-learning models could improve postoperative prognoses in daily clinical practice.

This study has the following hypotheses 1) patients might have a high-risk of delirium after knee arthroplasty and long hospital stay, and 2) postoperative delirium can be predicted through machine-learning using only preoperative features. Thus, the objectives of this study were: 1) to identify key preoperative risk factors, especially modifiable factors, for delirium development, and 2) to develop and validate a machine-learning model for predicting postoperative delirium in knee arthroplasty patients.

## Methods

### Study population

This study included the patient who underwent primary and revision knee arthroplasty from January 2016 to Sep 2019 at two tertiary referral hospitals [15]. The developmental cohort included patients from one hospital, and the validation cohort included patients from another. The type of knee arthroplasty was defined as follows: Unilateral knee arthroplasty (UKA), Total knee arthroplasty (TKA), and revision knee arthroplasty. Exclusion criteria were 1) those who were younger than 50 years, 2) those who had established an active delirium at the time of hospitalization [16, 17]. Lowering the age threshold may compromise the integrity of cohort by including patients with underlying disease like bone tumor, RA, osteonecrosis, etc. Increasing the threshold may inefficiently shrink the study population. Fifty years age threshold was set by rule of thumb.

**Fig. 1:**
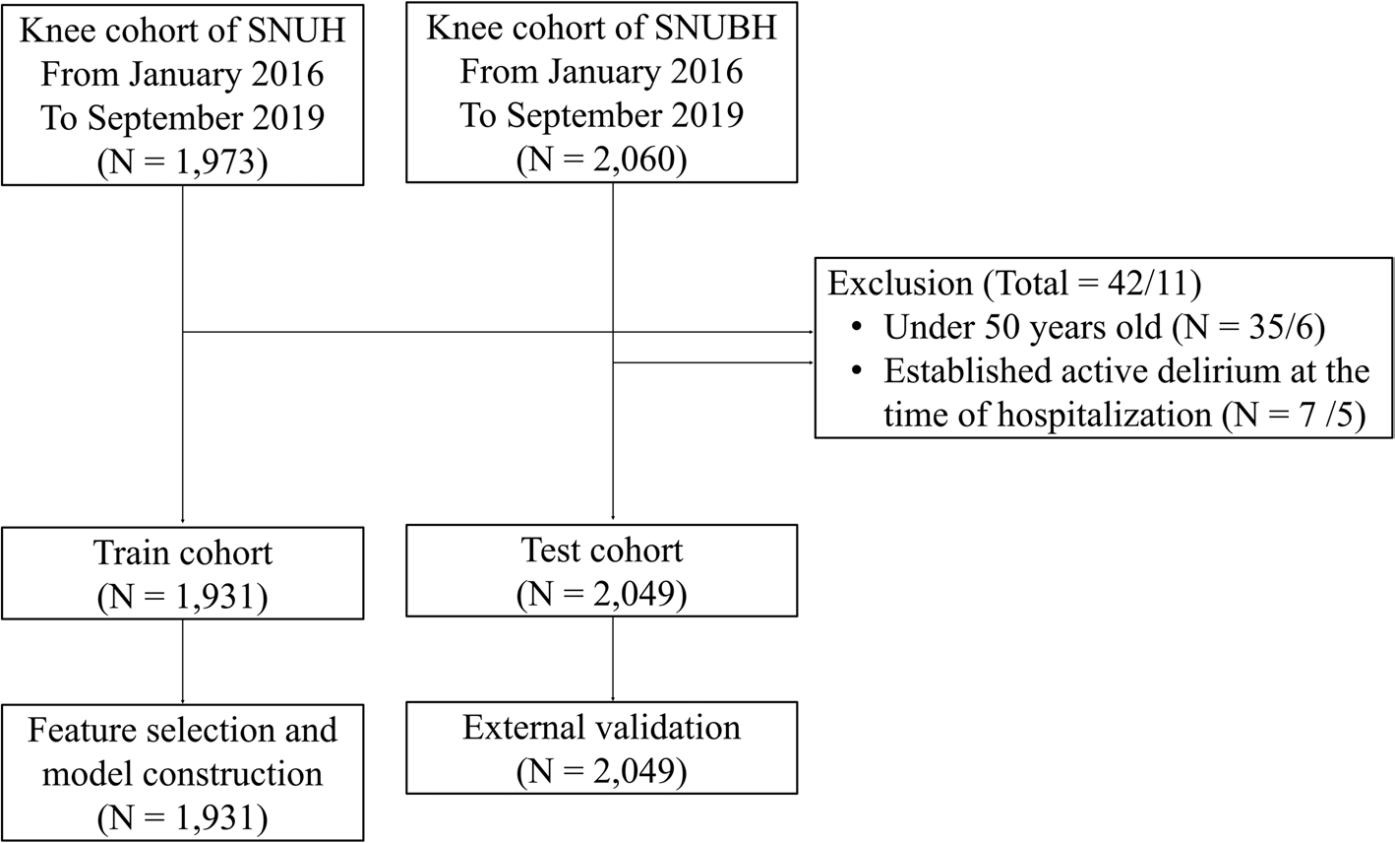
The study population. A total of 1,931 and 2,049 patients from two tertiary teaching hospitals were included in the analysis

A total of 4,029 patients were eligible (1,973 from hospital A and 2,060 from hospital B). After apply the exclusion criteria, 1,931 patients from hospital A were assigned to the developmental cohort and 2,049 patients from hospital B were assigned to the validation cohort (Fig. 1). Baseline characteristics of both cohorts are listed in Table 1. The mean age was 71.0 (standard deviation [SD]: 6.9) years in the development cohort and 71.3 (SD: 6.7) in the validation cohort. Female comprised 86% in the development cohort and 88% in the validation cohort.

**Table 1:** Baseline characteristics of the developmental and validation cohorts

Characteristics	**Developmental cohort (** ***N*** ** = 1,931)**		**Validation cohort (** ***N*** ** = 2,049)**		*P-*value
	Value	Missing	Value	Missing	
Age (SD)	71.0 (6.9)	-	71.3 (6.7)	-	0.26
Sex					
M	269 (14%)	-	241 (12%)	-	0.041
F	1,662 (86%)		1808 (88.0%)		
BMI (SD)	26.8 (3.7)	46	27.1 (3.6)	-	0.025
Type of surgery					
UKA	90 (4.7%)	-	12 (0.6%)	-	< 0.001
TKA	1,663 (86.1%)		1,907 (93.1%)		
Revision knee arthroplasty	178 (9.2%)		130 (6.3%)		
Operation numbers					
1	1,261 (65.3%)	-	1,404 (68.5%)	-	0.03
2	670 (34.7%)		645 (31.5%)		
Type of anesthesia					
General	149 (7.7%)	47 (2.4%)	18 (0.9%)	10 (0.5%)	< 0.001
Spinal or Epidural	1,735 (89.8%)		2021 (98.6%)		
Fall-down risk					
High	262 (13.6%)	433 (22.4%)	197 (9.6%)	-	< 0.001
Low	1236 (64.0%)		1,852 (90.4%)		
Number of anticholinergic cognitive drugs (SD)	0.11 (0.37)	-	0.09 (0.34)	-	0.15
Number of hypnotics and sedatives drugs (SD)	0.16 (0.46)	-	0.18 (0.51)	-	0.16
Total number of drugs (SD)	5.7 (4.2)	-	5.2 (4.3)	-	0.001
Visual impairment					
Y	34 (1.8%)	16 (0.8%)	150 (7.3%)	9 (0.4%)	< 0.001
N	1,881 (97.4%)		1,890 (92.2%)		
Hearing impairment					
Y	88 (4.6%)	16 (0.8%)	160 (7.8%)	-	< 0.001
N	1,827 (94.6%)		1,889 (92.2%)		
Neurologic disorders (dementia, Parkinson’s disease, epilepsy, headache disorder)					
Y	526 (27.2%)	-	432 (21.1%)	-	< 0.001
N	1,405 (72.8%)		1,617 (78.9%)		
Depression					
Y	120 (6.2%)	-	104 (5.1%)	-	0.12
N	1,811 (93.8%)		1,945 (94.9%)		

*UKA* Unicompartment knee arthroplasty, *TKA* Total knee replacement arthroplasty, *SD* Standard deviation, *BMI* Body mass index

### Surgical protocol

Both cohorts were treated via either a parapatellar or mid-vastus approach depending on surgeons’ preferences [15]. A posteriorly stabilized implant was placed in more than 80% of TKA cases. One gram of intra-articular tranexamic acid (TXA) was given unless patients had the following contraindications: TXA allergy, a history of deep vein thrombosis, pulmonary embolism, or ischemic heart or cerebrovascular disease, and/or a glomerular filtration rate (GFR) less than 60 mL/min [15]. Pain was controlled by Celecoxib 200 mg bid, Tramadol 37.5 mg tid from 1 day before to 1 week after the surgery and IV PCA (nefopam 80mcg, fentanyl 1000mcg) for 3 days after surgery. Continuous passive motion (CPM) was applied 1 day after the surgery [15]. Ambulation was permitted 12 h after the surgery [15]. Periarticular multimodal drug injection (Ropivacaine 225 mg + Ketorolac 30 mg) was applied at 1 day after the surgery.

### Outcomes

Primary outcomes were the development of delirium during the first postoperative week. Delirium assessment was conducted retrospectively using EHRs.

First, diagnosis of delirium was made based on the Diagnostic and Statistical Manual of Mental Disorder (DSM-5) [18]. Natural language words were collected from EHRs implicating the presence of delirium. Red flag word set included disturbance in attention, disoriented features, and behavioral alteration [19, 20]. A total of 69 natural language words were included. Second, two medical doctors reviewed postoperative delirium consultation with psychiatrists and postoperative antipsychotic drugs prescription records. Findings were adjudicated by a psychiatrist who had extensive training in delirium assessment [15].

When psychiatrist’s diagnosis was absent, diagnosis of delirium should be conducted in more complicated method. To be more specific, those red flag word set contains “increased irritability, no motor response, no verbal response, aggressive behavior, delusional, inappropriate emotional response, hard to communicate, lose orientation to time, place, person, decreased attention”. Inside EHR, not only physician but nurses also record patients’ status in timely fashion. Thus, it contains pretty much comprehensive and thorough information pertaining to patients’ medical and mental condition. Therefore, records containing those words in red flag set cannot directly diagnose but can assist psychiatrist determine whether patients were delirious at that time or not. For example, when red flag words were present on some patient’s EHR record and antipsychotic drugs were prescribed on the same day, the patient was very likely to be delirious. Otherwise, when antipsychotic drugs were not prescribed, we analyzed entire EHR record of each patient and decision was made in a most conservative manner. To sum up, patients without enough evidence of delirium was classified as non-delirium and there was no missing data relating to the outcome measurement.

Therefore, delirium assessment was conducted by trained staffs using DSM-5 augmented with a validated medical record review method [15, 19–21].

### Predictor variables

A total of 63 variables were initially chosen as candidate predictors based on findings of previous studies [5, 6, 8, 10, 15, 21, 22]. They are listed in Additional file 1. After removing variables with which less than 10 patients present non-missing values, 54 variables remained. These variables were directly compared with the final outcome value; postoperative delirium; and variables with *p*-value < 0.05 were only chosen, which made them 20. When calculating *p*-value, t-test and chi-square test were used in continuous and categorical variables, respectively. These 20 variables are listed in Table 2.

**Table 2:** Comparison of the delirium and non-delirium groups of the developmental cohort

Characteristics	**Developmental cohort (** ***N*** ** = 1,931)**				
	Delirium (*N* = 111, 5.7%)	Non-delirium (*N* = 1,820)	Total	*p-*value	Odds ratio (95% CI)
Selected key variables					
Age (SD)	76.4 (6.2)	70.7 (6.8)	71.0 (6.9)	< 0.001	1.15 (1.11–1.19)
Albumin (SD)	4.0 (0.3)	4.1 (0.3)	4.1 (0.3)	0.035	0.55 (0.31–0.96)
Number of hypnotics and sedatives drugs (SD)	0.29 (0.62)	0.15 (0.45)	0.16 (0.46)	0.024	1.58 (1.17–2.15)
Fall-down risk					
High	26 (29.9%)	236 (16.7%)	262 (17.5%)	0.002	2.1 (1.3–3.4)
Low	61 (70.1%)	1,175 (83.3%)	1,236 (82.5%)		
Total number of drugs (SD)	7.4 (5.1)	5.6 (4.1)	5.7 (4.2)	< 0.001	1.09 (1.05–1.13)
Neurologic disorders					
Y	51 (45.9%)	475 (26.1%)	526 (27.2%)	< 0.001	2.4 (1.6–3.5)
N	60 (54.1%)	1,345 (73.9%)	1,405 (72.8%)		
Depression					
Y	17 (15.3%)	103 (5.7%)	120 (6.2%)	< 0.001	3.0 (1.7–5.2)
N	94 (84.7%)	1,717 (94.3%)	1,811 (93.8%)		
Unselected variables					
Hearing impairment					
Y	11 (10.1%)	77(4.3%)	88 (4.6%)	0.005	2.5 (1.3–4.9)
N	98 (89.9%)	1,729 (95.7%)	1,827 (95.4%)		
The type of surgery					
UKA	2 (1.8%)	88 (4.8%)	90 (4.7%)	0.014	n.s
TKRA	91 (82.0%)	1,572 (86.4%)	1,663 (86.1%)		
Revision- TKRA	18 (16.2%)	160 (8.8%)	178 (9.2%)		
eGFR (MDRD) (SD)	73.7 (20.9)	81.6 (21.4)	81.2 (21.5)	< 0.001	0.98 (0.97–0.99)
Sodium (SD)	140.5 (2.6)	141.0 (2.3)	141.0 (2.4)	0.032	0.92 (0.86–0.99)
Anticholinergic cognitive drugs burden (SD)	0.22 (0.49)	0.10 (0.36)	0.11 (0.37)	0.018	2.28 (1.76–2.95)
Obstructive sleep apnea					
Y	3 (2.7%)	8 (0.4%)	11 (0.6%)	0.022	n.s
N	108 (97.3%)	1,812 (99.6%)	1,920 (99.4%)		
Diabetic mellitus					
Y	45 (40.5%)	504 (27.7%)	549 (28.4%)	0.004	1.78 (1.20–2.64)
N	66 (59.5%)	1,316 (72.3%)	1,382 (71.6%)		
AKI					
Y	4 (3.6%)	15 (0.8%)	19 (1.0%)	0.020	n.s
N	107 (96.4%)	1,805 (99.2%)	1,912 (99.0%)		
Atrial fibrillation					
Y	13 (11.7%)	96 (5.3%)	109 (5.6%)	0.004	2.38 (1.29–4.40)
N	98 (88.3%)	1,724 (94.7%)	1,822 (94.4%)		
Ischemic heart disease					
Y	33 (29.7%)	365 (20.1%)	398 (20.6%)	0.014	1.69 (1.10–2.57)
N	78 (70.3%)	1,455 (79.9%)	1,533 (79.4%)		
Cerebrovascular disease					
Y	29 (26.1%)	272 (14.9%)	301 (15.6%)	0.002	2.01 (1.29–3.13)
N	82 (73.9%)	1,548 (85.1%)	1,630 (84.4%)		
Peripheral arterial disease					
Y	24 (21.6%)	134 (7.4%)	158 (8.2%)	< 0.001	3.47 (2.14–5.64)
N	87 (78.4%)	1,686 (92.6%)	1,773 (91.8%)		
Septic arthritis					
Y	12 (10.8%)	107 (5.9%)	119 (6.1%)	0.036	1.94 (1.03–3.64)
N	99 (89.2%)	1,713 (94.1%)	1,812 (93.8%)		

*UKA* Unicompartment knee arthroplasty, *TKRA* Total knee replacement arthroplasty, *Revision-TKRA* Revision total knee replacement arthroplasty, *AKI* Acute kidney injury, *SD* Standard deviation, *BMI* Body mass index, *eGFR* Estimated glomerular filtration rate

Demographic data included age, sex, body mass index (BMI), current smoker, and alcohol consumption (more than 5 times/week) [23, 24]. The American Society of Anesthesiologists Classification (ASA Class), fall-down risk, visual impairment, hearing impairment, and sleep impairment were extracted from preoperative checklists. Morse Fall Scale (MFS) was used for fall-down risk (Table 3) [25]. Score was calculated based on the following six patient factors: previous fall down history, presence of secondary diagnosis, usage of walking assistant device, presence of heparin lock, stability of ambulation, and psychiatric condition. The fall-down risk variable was defined as dichotomization of the final risk score. The type of surgery, operation number, and type of anesthesia (general or spinal or epidural) were included. Type of surgery included unilateral knee arthroplasty, total knee arthroplasty, revision total knee arthroplasty. Operation number included unilateral, simultaneous bilateral, staged bilateral (1-week interval).

**Table 3:** Morse fall risk assessment

Risk Factor	Scale	Score
History of Falls	Yes	25
	No	0
Secondary Diagnosis	Yes	15
	No	0
Ambulatory Aid	Furniture	30
	Crutches/ Cane/ Walker	15
	None / Bed Rest / Wheel Chair / Nurse	0
IV / Heparin Lock	Yes	20
	No	0
Gait / Transferring	Impaired	20
	Weak	10
	Normal / Bed Rest / Immobile	0
Mental status	Forgets Limitations	15
	Oriented to Own Ability	0

Morse Fall Score

High Risk: 45 and higher

Low Risk: 0–44

*IV* Intra Venous

Serum laboratory results included blood urea nitrogen (BUN), creatinine, BUN/Cr ratio, eGFR (Modification of Diet in Renal Disease), hemoglobin (Hb), hematocrit (Hct), white blood cell (WBC), c-reactive protein (CRP), erythrocyte sedimentation rate (ESR), total protein, albumin, prothrombin time (INR), ALP, AST, ALT, total bilirubin, total cholesterol, sodium, and potassium (the latest value within 90 days before surgery). Urine laboratory results included albumin level (the latest value within 90 days before surgery). The collection of preoperative laboratory values is a routine procedure in most Korean hospitals. Thus, missing values were quite rare.

To explore preoperative medication status and underlying diseases, admission records were combined with in-hospital drug prescriptions [12]. Three important drug classes (i.e., anticholinergic drugs, hypnotics/sedatives, and opioids) were included in the analyses. Drug categorization was based on the Anatomical Therapeutic Chemical (ATC) classification. Drug details are listed in Additional file 2. Anticholinergic drug cognitive burden scale [26], number of hypnotics and sedatives drugs, number of opioid drugs, and total number of drugs (any kinds of oral medication) were extracted to represent preoperative medication status.

Hypertension, diabetes mellitus, hypoglycemia, hypercholesterolemia, acute kidney injury, end-stage renal disease, atrial fibrillation, pulmonary embolism, ischemic heart disease, neurologic disorders (Parkinson’s disease, dementia, epilepsy, headache disorder), depression, generalized anxiety disorder, schizoaffective disorder, obstructive sleep apnea, cerebrovascular disease, meningitis, adrenal insufficiency, peripheral arterial disease, peripheral vascular disease, malignancy, sepsis, septic arthritis, and HIV + were extracted. History of trauma and history of amputation were also extracted.

### Statistical analyses

**Fig. 2:**
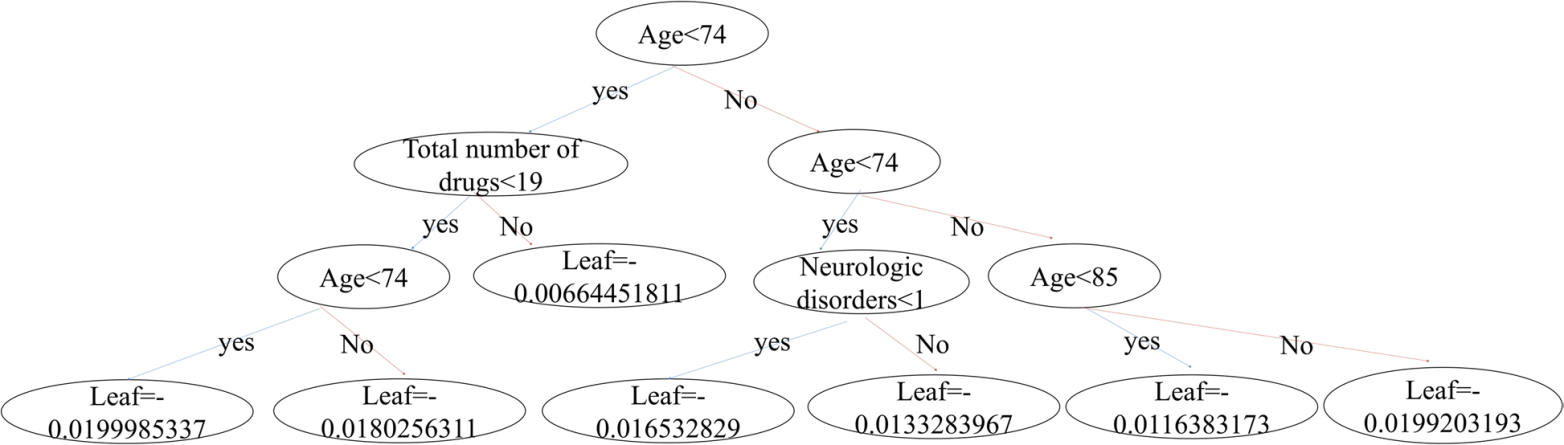
Visualization of one of gradient boosting trees [13]

All statistical analyses were performed using SAS version 9.4 (SAS Inc, Cary, NC, USA). A gradient boosting machine (GBM) was used to predict the probability of delirium, employing all predictor variables. GBM used a series of decision trees, where each tree corrected residuals of previous trees. XGboost is a machine learning based gradient boosting model. Figure 2 is the visualization of one of 300 trees that consists our final model. Tree leaf on the lower end is the prediction score of each tree. On each step, tree is constructed with proper split values. Addition of extra branch on each tree may enhance the accuracy of the model while increasing the complexity of the model. These accuracy score and complexity score are quantified to determine the necessity of extra tree-branching. Following this particular method, XGBoost became a compelling technique in machine learning that learns fast while avoiding overfitting. More details can be found at https://arxiv.org/abs/1603.02754. AUROC was obtained by roc_auc_score function in scikit-learn library. It is calculated as area under the curve when FPR(false positive ratio) and TPR(true positive ratio) are plotted on XY axis with varying threshold values. Python 3.7.11 and Google Colaboratory were used to encode the machine-learning algorithm. Missing values were imputed using a built-in GBM algorithm. Two feature-selection methods were used: sequential feature selection and forward elimination. The stratified K-fold (K = 10) approach was used to select predictor variables and optimize hyperparameters.

The developmental cohort was divided into two subgroups: a training group (*N* = 1351, 70%) and a test group (*N* = 580, 30%). The 20 variables were divided into categorical variables and continuous variables. Feature selection method with stratified tenfold cross validation on training group was applied for each variable subgroup. Top three variables selected by this algorithm were included in the final model. For each variable subgroup, the 4^th^, 5^th^ and 6^th^ ranked variables presented by this algorithm were tested in conjunction with six pre-selected variables. The variable that maximized the performance of internal validation was incorporated into the final model. The final model contained a total of seven variables.

The final model was trained using the training group (*N* = 1351) with seven selected variables and tested with the test group (*N* = 580) to calibrate the internal validation within the developmental cohort. Youden index was used to identify the optimal ROC curve threshold [27]. External validation was performed using all data from one institution as a test set (*n* = 2,049).

## Results

**Fig. 3:**
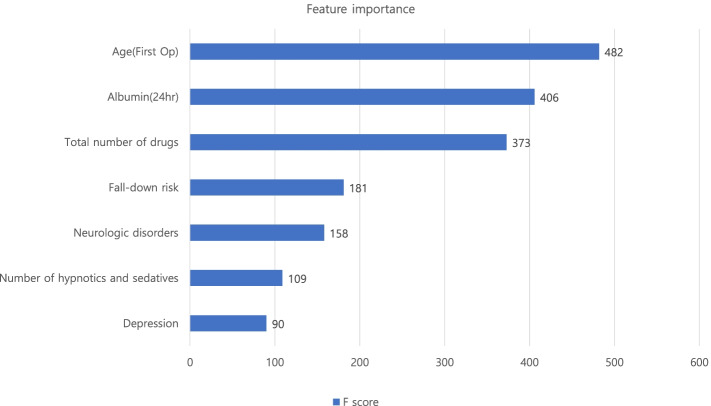
The importance factor of the complete model. The feature importance plot was shown from the highest F score. The feature’s higher F score have a greater impact on the prediction of postoperative delirium

**Fig. 4:**
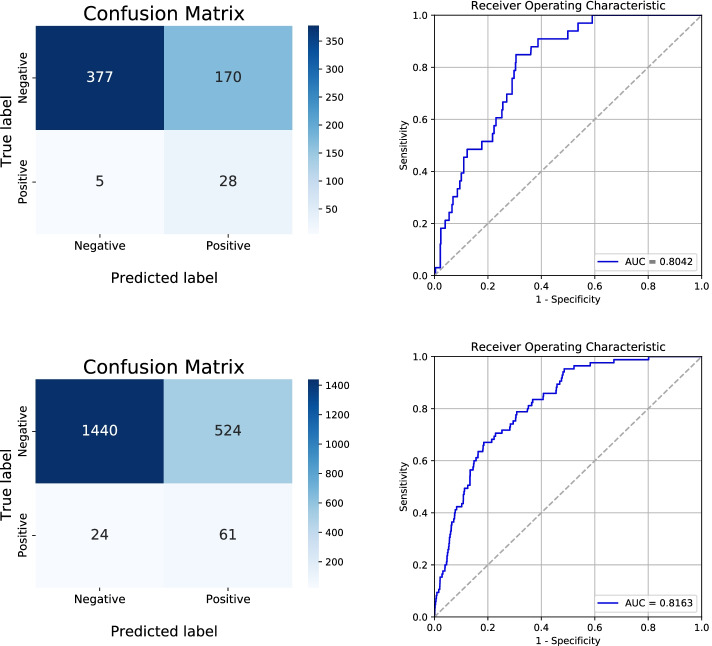
The AUROC and confusion table of the model. The pictures on the left from the top to the bottom are the AUROC curve of the internal and external validation, respectively. The pictures on the right from the top to the bottom are the confusion table after internal and external validation, respectively

Of 3,980 patients, 196 (4.9%) were diagnosed with delirium after knee arthroplasty. These delirious patients had longer hospital stays (15.4 days vs. 11.6 days, *p* < 0.001) than non-delirious patients. Of 20 variables, seven key predictors were selected for the model, including four continuous variables (age, serum albumin level, number of hypnotics and sedatives drugs, and total number of drugs) and three categorial variables (neurologic disorders, depression, and fall-down risk). The odds ratio of polypharmacy (total number of drugs >  = 6) patients was 2.38 (95% confidence interval (CI): 1.55–3.30). The XGBoost importance plot is shown in Fig. 3 [12]. The performance of the final model on developmental cohort calibrated as AUC was 0.80 (95% CI: 0.77–0.84) after internal validation. Optimal threshold, sensitivity and specificity of the model was 0.085, 0.85, 0.69 respectively. For validation cohort, AUC score was 0.82 (95% CI: 0.80 – 0.83) and the sensitivity, specificity was 0.72 and 0.73 respectively. Our model was uploaded in an online website, which can be found at https://safetka.connecteve.com. The model automatically calculates the probability of postoperative delirium and visualize the value itself along with weights and significance hierarchy of each seven variables that users enter. The model was saved as “Predict_Delirium_after_knee_arthroplasty.pkl”. Thus, clinicians could still use the model when several value of variables cannot be obtained. The detailed protocol was uploaded in github repository; https://github.com/JasonJeongMed/Postoperative_Delirium_prediction_afterKA. The AUROC curve and the confusion table of internal and external validation are shown in Fig. 4.

## Discussion

In the present study, the postoperative delirium risk was predicted based on the preoperative EHRs data using a machine-learning algorithm. Thus, this algorithm can be used not only to screen high-risk group, but also to assist orthopedic surgeons to take more proactive approach on delirium prevention [12].

This algorithm can also be applied in independent institutions, because the predictive performance could be maintained in an external validation. Key preoperative variables to predict delirium after knee arthroplasty were incorporated into a machine-learning algorithm. The model yielded sound performance in terms of AUC in both internal and external validation, with comparable sensitivity and specificity values respectively. Thus, the model is not institution-specific. It can be readily accessed in the outpatient clinic [12]. Even when several variables were not able to obtain, given that XGboost model works with missing values, the physician could still use this model with the rest of the variables. Twenty-one (10.7%) patients in the delirious group were hospitalized for more than 3 weeks, leading to high costs of care. Thus, implying that presence of delirium and long hospital stay may be correlated. However, direct causality cannot be guaranteed. Similar causal relationship issue regarding modifiable variables is also addressed below.

Several previous studies have used machine-learning to develop delirium prediction models [14, 24]. However, these models were not validated with an external cohort [14, 24]. Corradi et al. have developed a model using a large dataset (128 variables) with high ROC-AUC (91%) without an external validation for model [24]. In addition, too many variables can compromise an external validation. Our machine-learning model used only seven key variables that appeared to be the most important factors with respect to correlation with delirium. All variables were commonly measured in a clinical setting. Our model was not only internally validated, but also externally validated with patients from an independent institution, confirming that our model was not overfitted. Thus, its application to other institutions can be warranted.

**Fig. 5:**
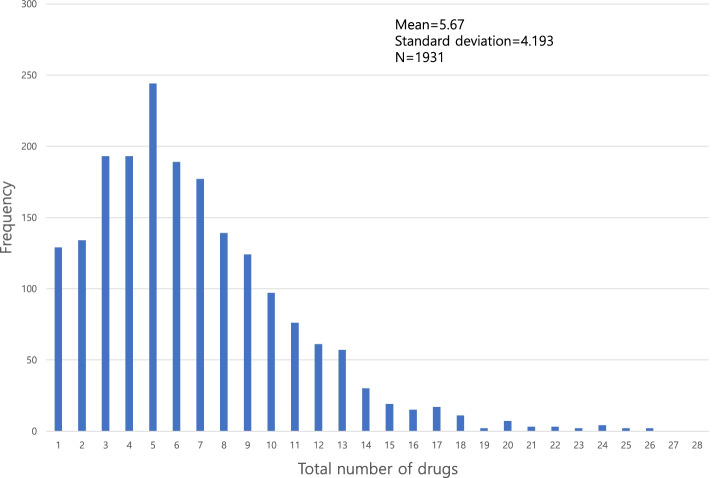
Distribution of total number of drugs at admission. A total of 1,931 patients took average 5.67 pills (SD: 4.19)

Key preoperative features included in our model have already been discussed in previous studies as risk factors of delirium. Inouye et al. have described that age, fall-down risk, neurologic disorders, depression, and polypharmacy are risk factors of delirium [28]. Suman et al. described that those with lower albumin level and lower nutrition status have far higher risk for delirium using pooled analysis [29]. In our study, pre-operative patients take an average 5.75 drugs (Fig. 5). John et al. have established a prediction model, with polypharmacy having a high rank [24]. In our study, the odds ratio of those with polypharmacy (total number of drugs >  = 6) having delirium was 2.38 (95% CI: 1.55–3.30).

Our work had several limitations. First, the presence of delirium was not based on structured methods such as Mini-Mental State Examination (MMSE), CAM-ICU, and CERAD (Consortium to Establish a Registry for Alzheimer’s Disease) neuropsychological battery. Chart based retrospective decision of delirium can omit several patients with delirium when chart information is not sufficient to prove the status of the patients. This could lead to relatively low proportion of delirious patients compared to other studies that focused on delirium prediction [6, 30, 31]. However, various methods including consultation with psychiatrist, prescription of antipsychotic drug, and natural language analysis from medical records were deployed to detect postoperative delirium. Moreover, two psychiatrists directly reviewed medical records to confirm delirium cases to enhance accuracy. Secondly, this algorithm was developed by one medical center. It failed to cover the general population. Substantially higher female proportion of woman (> 80%) compared to western knee arthroplasty recipients (around 60%) shows that demographics may vary by institutions and countries [1, 32, 33]. Although our model was based on one particular institution, numbers of cases in both developmental and validation cohorts were larger than those of other studies. Thirdly, only patients with knee arthroplasty were recruited in the first place. This might have compromised the generalizability of the model when applying to general patients. However, incidence of postoperative delirium varies by type of surgical intervention the patient went through. Thus, by limiting the population to only single surgical intervention recipient, the machine learning model could efficiently highlight the rest of the variables that can contribute to incident delirium after surgery. Fourthly, patient’s underlying history was only identified as the name of the disease itself. It failed to deliver the severity and functional impairment of patients. However, incorporation of the severity index could seriously compromise the simplicity of EHRs information. Furthermore, we collected multiple disease histories with varying comorbidities. These variables could putatively replace the severity index of particular diseases. Lastly, modifiable variables we included in the model (albumin level, number of drugs taken preoperatively) do not actually guarantee that modification of such variables will reduce the incidence of delirium. Especially recommendation on our web-app was based on reduction of the possibility of postoperative delirium. Thus, clinical evidence of impact of these variables on postoperative delirium on real hospital setting has yet to be established. Confounders that affect both postoperative delirium and modifiable variables should be taken under consideration to clarify the causal relationship. However, we suggest that boosting the low albumin value (especially lower than 3.0 mg/dl) and refrain from taking unnecessary duplicated medication are considered as routine procedures in several hospitals. Moreover, studies described these two variables as potential risk factors for delirium [24, 28, 29]. Thus, even clinical evidence lacks, physicians can take this modification as a precautionary measure when the model recommends as such. We currently embarked on a prospective study with delirium prediction including treatment response modeling to investigate potential risk factors that has real impact on postoperative delirium. We expect that this follow-up study will verify the causal mechanism that links modifiable factors we suggested and postoperative delirium.

## Conclusion

With just 7 preoperative variables, a web-based machine learning algorithm that can predict delirium after knee arthroplasty was constructed. The model is simple. It was validated to improve both short- and long-term prognoses of knee arthroplasty patients. Postoperative delirium could be a potential correlating factor of longer hospital stay. Thus surgeons should strive to avoid.

## Supplementary Information

**Additional file 1: Supplementary Table 1.** Comparison of the Delirium and non-delirium groups of the developmental cohort in 64 features.

**Additional file 2: Supplementary Table 2.** Keywords for classification of medication.

## Data Availability

The datasets used and/or analysed during the current study are available from the corresponding author on reasonable request.

## Abbreviations

UKA: Unicompartment knee arthroplasty
TKA: Total knee arthroplasty
RKA: Revision knee arthroplasty
TXA: Tranexamic acid
CPM: Continuous passive motion
SFS: Sequential feature selection
XGBoost: Extreme Gradient Boosting algorithm
GBM: Gradient boosting machine
EHRs: Electronic health records
DSM-5: Diagnostic and statistical manual of mental disorder
CAM-ICU: Confusion assessment method-intensive care unit
MMSE: Mini-mental state examination
CERAD: Consortium to establish a registry for alzheimer’s disease
AUC: Area under curve
CI: Confidence interval
SD: Standard deviation
