# Supplementary material for: A machine-learning model to predict postoperative delirium following knee arthroplasty using electronic health records
Source: BMC Psychiatry. 2022 Jun 27;22:436. doi: 10.1186/s12888-022-04067-y (PMC9235137; doi:10.1186/s12888-022-04067-y)
Supplement: Supplementary file 1 — Additional file 1: Supplementary Table 1. Comparison of the Delirium and non-delirium groups of the developmental cohort in 64 features. [file 12888_2022_4067_MOESM1_ESM.docx]

Supplementary Table 1. Comparison of the Delirium and non-delirium groups of the developmental cohort in 64 features

| Characteristics | | | **Developmental cohort (N = 1,931)** | | | | |
| --- | --- | --- | --- | --- | --- | --- | --- |
|  |  |  | Delirium  (N = 111, 5.7%) | Non-delirium  (N = 1,820) | Total | *p-*value | Odds ratio  (95% CI) |
| Selected key variables | Age (SD) |  | 76.4 (6.2) | 70.7 (6.8) | 71.0 (6.9) | <0.001 | 1.15 (1.11 – 1.19) |
|  | Albumin (SD) |  | 4.0 (0.3) | 4.1 (0.3) | 4.1 (0.3) | 0.035 | 0.55 (0.31 – 0.96) |
|  | Number of hypnotics and sedatives drugs (SD) |  | 0.29 (0.62) | 0.15 (0.45) | 0.16 (0.46) | 0.024 | 1.58 (1.17 – 2.15) |
|  | Fall-down risk | High | 26 (29.9%) | 236 (16.7%) | 262 (17.5%) | 0.002 | 2.1 (1.3 – 3.4) |
|  |  | Low | 61 (70.1%) | 1,175 (83.3%) | 1,236 (82.5%) |  |  |
|  | Total number of drugs (SD) |  | 7.4 (5.1) | 5.6 (4.1) | 5.7 (4.2) | <0.001 | 1.09 (1.05 – 1.13) |
|  | Neurologic disorders | Y | 51 (45.9%) | 475 (26.1%) | 526 (27.2%) | <0.001 | 2.4 (1.6 – 3.5) |
|  |  | N | 60 (54.1%) | 1,345 (73.9%) | 1,405 (72.8%) |  |  |
|  | Depression | Y | 17 (15.3%) | 103 (5.7%) | 120 (6.2%) | <0.001 | 3.0 (1.7 – 5.2) |
|  |  | N | 94 (84.7%) | 1,717 (94.3%) | 1,811 (93.8%) |  |  |
| Unselected variables | Sex | Male | 18 (16.2%) | 251 (13.8%) | 269 (13.9%) | 0.47 | 1.2 (0.7 – 2.0) |
|  |  | Female | 93 (83.8%) | 1,569 (86.2%) | 1,662 (86.1%) |  |  |
|  | BMI (SD) |  | 26.6 (3.5) | 26.8 (3.7) | 26.8 (3.7) | 0.54 | 0.98 (0.93 – 1.04) |
|  | Current smoker | Y | 3 (2.8%) | 43 (2.4%) | 46 (2.4%) | 0.74 | n.s |
|  |  | N | 105 (97.2%) | 1,763 (97.6%) | 1,868 (97.6%) |  |  |
|  | Alcohol consumption | High | 7 (6.5%) | 117 (6.5%) | 124 (6.5%) | >0.99 | 1.0 (0.45 – 2.20) |
|  |  | Low | 101 (93.5%) | 1,689 (93.5%) | 1,790 (93.5%) |  |  |
|  | ASA class | 1 | 22 (20.6%) | 343 (19.0%) | 365 (19.0%) | 0.36 | (reference) |
|  |  | 2 | 79 (73.8%) | 1,291 (71.3%) | 1,370 (71.5%) |  | 1.1 (0.7 – 1.8) |
|  |  | 3+ | 6 (5.6%) | 176 (9.7%) | 182 (9.5%) |  |  |
|  | Visual impairment | Y | 1 (0.9%) | 33 (1.8%) | 34 (1.8%) | 0.72 | n.s |
|  |  | N | 108 (99.1%) | 1,773 (98.2%) | 1,881 (98.2%) |  |  |
|  | Hearing impairment | Y | 11 (10.1%) | 77(4.3%) | 88 (4.6%) | 0.005 | 2.5 (1.3 – 4.9) |
|  |  | N | 98 (89.9%) | 1,729 (95.7%) | 1,827 (95.4%) |  |  |
|  | Sleep impairment | Y | 10 (11.5%) | 92 (6.5%) | 102 (6.8%) | 0.075 | 1.9 (0.9 – 3.7) |
|  |  | N | 77 (88.5%) | 1,315 (93.5%) | 1,392 (93.2%) |  |  |
|  | Type of surgery | UKA | 2 (1.8%) | 88 (4.8%) | 90 (4.7%) | 0.014 | n.s |
|  |  | TKRA | 91 (82.0%) | 1,572 (86.4%) | 1,663 (86.1%) |  |  |
|  |  | Revision- TKRA | 18 (16.2%) | 160 (8.8%) | 178 (9.2%) |  |  |
|  | Operation numbers | 1 | 71 (64.0%) | 1,190 (65.4%) | 1,261 (65.3%) | 0.76 | 0.94 (0.63 – 1.40) |
|  |  | 2 | 40 (36.0%) | 630 (34..6%) | 670 (34.7%) |  |  |
|  | Type of anesthesia | General | 9 (8.5%) | 140 (7.9%) | 149 (7.9%) | 0.82 | 1.1 (0.54 – 2.20) |
|  |  | Spinal or Epidural | 97 (91.5%) | 1,638 (92.1%) | 1,735 (92.1%) |  |  |
|  | BUN (SD) |  | 18.7 (6.3) | 18.1 (6.2) | 18.2 (6.2) | 0.37 | 1.01 (0.99 – 1.04) |
|  | Creatinine (SD) |  | 0.86 (0.28) | 0.80 (0.43) | 0.80 (0.42) | 0.15 | 1.23 (0.91 – 1.66) |
|  | BUN/Cr ratio (SD) |  | 22.4 (6.7) | 23.7 (6.8) | 23.6 (6.8) | 0.052 | 0.97 (0.94 – 1.00) |
|  | eGFR (MDRD) (SD) |  | 73.7 (20.9) | 81.6 (21.4) | 81.2 (21.5) | <0.001 | 0.98 (0.97 – 0.99) |
|  | Hb (SD) |  | 12.7 (1.7) | 12.7 (1.3) | 12.7 (1.3) | 0.67 | 0.96 (0.83 – 1.11) |
|  | Hct (SD) |  | 38.3 (4.4) | 38.4 (3.8) | 38.4 (3.8) | 0.72 | 0.99 (0.94 – 1.04) |
|  | WBC (SD) |  | 6.7 (2.4) | 6.4 (1.8) | 6.4 (1.8) | 0.25 | 1.07 (0.98 – 1.18) |
|  | CRP (SD) |  | 0.58 (2.61) | 0.30 (1.20) | 0.32 (1.32) | 0.28 | 1.08 (1.00 – 1.18) |
|  | ESR (SD) |  | 20.4 (17.3) | 20.5 (16.2) | 20.5 (16.3) | 0.97 | 1.00 (0.98 – 1.02) |
|  | Total protein (SD) |  | 6.9 (0.4) | 7.0 (0.5) | 7.0 (0.5) | 0.26 | 0.80 (0.56 – 1.17) |
|  | PT(INR) (SD) |  | 0.98 (0.09) | 0.96 (0.07) | 0.96 (0.07) | 0.051 | 14.48 (1.74 – 120.81) |
|  | ALP (SD) |  | 72.0 (20.1) | 71.8 (24.4) | 71.8 (24.2) | 0.93 | 1.00 (0.99 – 1.01) |
|  | AST (SD) |  | 23.6 (9.9) | 23.5 (9.7) | 23.5 (9.7) | 0.94 | 1.00 (0.98 – 1.02) |
|  | ALT (SD) |  | 20.2 (13.8) | 21.0 (12.9) | 20.9 (13.0) | 0.54 | 1.00 (0.98 – 1.01) |
|  | Total bilirubin (SD) |  | 0.59 (0.22) | 0.58 (0.24) | 0.58 (0.24) | 0.72 | 1.17 (0.46 – 2.96) |
|  | Total cholesterol (SD) |  | 173.1 (45.5) | 178.6 (37.1) | 178.3 (37.6) | 0.34 | 1.00 (0.99 – 1.00) |
|  | Sodium (SD) |  | 140.5 (2.6) | 141.0 (2.3) | 141.0 (2.4) | 0.032 | 0.92 (0.86 – 0.99) |
|  | Potassium (SD) |  | 4.3 (0.4) | 4.3 (0.4) | 4.3 (0.4) | 0.75 | 1.08 (0.66 – 1.77) |
|  | Urine albumin | - | 67 (67.0%) | 1311 (76.0%) | 1378 (75.5%) | 0.14 | (reference) |
|  |  | +/- | 20 (20.0%) | 298 (17.3%) | 318 (17.4%) |  | 0.64 (0.42-0.98) |
|  |  | 1+ | 9 (9.0%) | 77 (4.5%) | 86 (4.7%) |  |  |
|  |  | 2++ | 4 (4.0%) | 38 (2.2%) | 42 (2.3%) |  |  |
|  | Anticholinergic cognitive drugs burden (SD) |  | 0.22 (0.49) | 0.10 (0.36) | 0.11 (0.37) | 0.018 | 2.28 (1.76 – 2.95) |
|  | Number of Opioid drugs (SD) |  | 0.86 (0.38) | 0.83 (0.44) | 0.83 (0.44) | 0.56 | 1.14 (0.73 – 1.79) |
|  | Schizoaffective disorder | Y | 2 (1.8%) | 2 (0.1%) | 4 (0.2%) | 0.018 | n.s |
|  |  | N | 109 (98.2%) | 1,818 (99.9%) | 1,927 (99.8%) |  |  |
|  | Pulmonary embolism | Y | 1 (0.9%) | 9 (0.5%) | 10 (0.5%) | 0.45 | n.s |
|  |  | N | 110 (99.1%) | 1,811 (99.5%) | 1,921 (99.5%) |  |  |
|  | Meningitis/Encephalitis | Y | 2 (1.8%) | 8 (0.4%) | 10 (0.5%) | 0.11 | n.s |
|  |  | N | 109 (98.2%) | 1,812 (99.6%) | 1,921 (99.5%) |  |  |
|  | Adrenal insufficiency | Y | 2 (1.8%) | 8 (0.4%) | 10 (0.5%) | 0.11 | n.s |
|  |  | N | 109 (98.2%) | 1812 (99.6%) | 1,921 (99.5%) |  |  |
|  | Obstructive sleep apnea | Y | 3 (2.7%) | 8 (0.4%) | 11 (0.6%) | 0.022 | n.s |
|  |  | N | 108 (97.3%) | 1,812 (99.6%) | 1,920 (99.4%) |  |  |
|  | HIV + | Y | 0 (0%) | 2 (0.1%) | 2 (0.1%) | n.s | n.s |
|  |  | N | 111 (100%) | 1818 (99.9%) | 1,929 (99.9%) |  |  |
|  | History of amputation | Y | 0 (0%) | 4 (0.2%) | 4 (0.2%) | n.s | n.s |
|  |  | N | 111 (100%) | 1,816 (99.8%) | 1,927 (99.8%) |  |  |
|  | Sepsis | Y | 0 (0%) | 8 (0.4%) | 8 (0.4%) | n.s | n.s |
|  |  | N | 111 (100%) | 1,812 (99.6%) | 1,923 (99.6%) |  |  |
|  | Generalized anxiety disorder | Y | 1 (0.9%) | 12 (0.7%) | 13 (0.7%) | 0.54 | n.s |
|  |  | N | 110 (99.1%) | 1,808 (99.3%) | 1,918 (99.3%) |  |  |
|  | Hypoglycemia | Y | 0 (0%) | 5 (0.3%) | 5 (0.3%) | n.s | n.s |
|  |  | N | 111 (100%) | 1,815 (99.7%) | 1,926 (99.7%) |  |  |
|  | Hypertension | Y | 84 (75.7%) | 1218 (66.9%) | 1,302 (67.4%) | 0.056 | 1.54 (0.99 – 2.40) |
|  |  | N | 27 (24.3%) | 602 (33.1%) | 629 (32.6%) |  |  |
|  | Diabetic mellitus | Y | 45 (40.5%) | 504 (27.7%) | 549 (28.4%) | 0.004 | 1.78 (1.20 – 2.64) |
|  |  | N | 66 (59.5%) | 1,316 (72.3%) | 1,382 (71.6%) |  |  |
|  | Hypercholesterolemia | Y | 45 (40.5%) | 683 (37.5%) | 728 (37.7%) | 0.53 | 1.14 (0.77 – 1.68) |
|  |  | N | 66 (59.5%) | 1,137 (62.5%) | 1,203 (62.3%) |  |  |
|  | AKI | Y | 4 (3.6%) | 15 (0.8%) | 19 (1.0%) | 0.020 | n.s |
|  |  | N | 107 (96.4%) | 1,805 (99.2%) | 1,912 (99.0%) |  |  |
|  | ESRD | Y | 8 (7.2%) | 101 (5.5%) | 109 (5.6%) | 0.46 | 1.32 (0.63 – 2.79) |
|  |  | N | 103 (92.8%) | 1,719 (94.5%) | 1,822 (94.4%) |  |  |
|  | Atrial fibrillation | Y | 13 (11.7%) | 96 (5.3%) | 109 (5.6%) | 0.004 | 2.38 (1.29 – 4.40) |
|  |  | N | 98 (88.3%) | 1,724 (94.7%) | 1,822 (94.4%) |  |  |
|  | Ischemic heart disease | Y | 33 (29.7%) | 365 (20.1%) | 398 (20.6%) | 0.014 | 1.69 (1.10 – 2.57) |
|  |  | N | 78 (70.3%) | 1,455 (79.9%) | 1,533 (79.4%) |  |  |
|  | O2 saturation (SD) |  | 95.5 (2.1) | 96.0 (2.1) | 96.0 (2.1) | 0.11 | 0.94 (0.86 – 1.02) |
|  | Cerebrovascular disease | Y | 29 (26.1%) | 272 (14.9%) | 301 (15.6%) | 0.002 | 2.01 (1.29 – 3.13) |
|  |  | N | 82 (73.9%) | 1,548 (85.1%) | 1,630 (84.4%) |  |  |
|  | Peripheral arterial disease | Y | 24 (21.6%) | 134 (7.4%) | 158 (8.2%) | <0.001 | 3.47 (2.14 – 5.64) |
|  |  | N | 87 (78.4%) | 1,686 (92.6%) | 1,773 (91.8%) |  |  |
|  | Peripheral venous disease | Y | 15 (13.5%) | 199 (10.9%) | 214 (11.1%) | 0.40 | 1.27 (0.72 – 2.24) |
|  |  | N | 96 (86.5%) | 1,621 (89.1%) | 1,717 (88.9%) |  |  |
|  | Malignancy | Y | 19 (17.1%) | 331 (18.2%) | 350 (18.1%) | 0.78 | 0.93 (0.56 – 1.54) |
|  |  | N | 92 (82.9%) | 1,489 (81.8%) | 1,581 (81.9%) |  |  |
|  | Septic arthritis | Y | 12 (10.8%) | 107 (5.9%) | 119 (6.1%) | 0.036 | 1.94 (1.03 – 3.64) |
|  |  | N | 99 (89.2%) | 1,713 (94.1%) | 1,812 (93.8%) |  |  |
|  | History of trauma | Y | 37 (33.3%) | 486 (26.7%) | 523 (27.1%) | 0.13 | 1.37 (0.91 – 2.06) |
|  |  | N | 74 (66.7%) | 1,334 (73.3%) | 1,408 (72.9%) |  |  |

* BMI: body mass index, SD: standard deviation, ASA class: American Society of Anesthesiologists, UKA: unicompartment knee arthroplasty, TKRA: total knee replacement arthroplasty, Revision-TKRA : revision total knee replacement arthroplasty, BUN: blood urea nitrogen, eGFR: estimated glomerular filtration rate, Hb: hemoglobin, Hct: hematocrit, CRP: c-reactive protein, ESR: erythrocyte sedimentation rate, PT: prothrombin time, ALP: alkaline phosphatase, AST: aspartate amino-transferase, ALT: alanine amino-transferase, HIV: human immunodeficiency virus, AKI: acute kidney injury, ESRD: end-stage renal disease
