# Supplementary material for: A machine-learning model to predict postoperative delirium following knee arthroplasty using electronic health records
Source: BMC Psychiatry. 2022 Jun 27;22:436. doi: 10.1186/s12888-022-04067-y (PMC9235137; doi:10.1186/s12888-022-04067-y)
Supplement: Supplementary file 2 — Additional file 2: Supplementary Table 2. Keywords for classification of medication. [file 12888_2022_4067_MOESM2_ESM.docx]

Supplementary Table 2. Keywords for classification of medication

| Anticholinergic | Hypnotics and sedatives | Opioid |
| --- | --- | --- |
| Amantadine | Alprazolam | Buprenorphine |
| Amitriptyline | Clonazepam | Fentanyl |
| Amoxapine | Diazepam | Oxycodone |
| Atropine | Etizolam | Tramadol |
| Benzotropine | Flurazepam | Tramadol/acetaminophen |
| Brompheniramine | Lorazepam |  |
| Carbamazepine | Oxazepam |  |
| Carbinoxamine | Triazolam |  |
| Chlorpheniramine | Zolpidem | |
| Chlorpromazine | |  |
| Clemastine | |  |
| Clomipramine | |  |
| Clozapine | |  |
| Cyclobenzaprine | |  |
| Darifenacin | |  |
| Desipramine | |  |
| Dicyclomine |  |  |
| Dimenhydrinate | |  |
| Doxepin |  |  |
| Flavoxate | |  |
| Hydroxyzine | |  |
| Hyoscyamine |  |  |
| Imipramine |  |  |
| Meclizine | |  |
| Meperidine | |  |
| Methocarbamol | |  |
| Nortriptyline | |  |
| Olanzapine | |  |
| Orphenadrine | |  |
| Oxacarbazepine | |  |
| Oxybutynin | |  |
| Indobufen | |  |
| Paroxetine |  |  |
| Perphenazine |  |  |
| Promethazine |  |  |
| Propantheline | |  |
| Quetiapine |  |  |
| Scopolamine |  |  |
| Thioridazine |  |  |
| Tolterodine | |  |
| Trifluoperazine |  |  |
| Trihexyphenidyl |  |  |
| Trimipramine |  |  |
